# Assessing the effect of hyperbaric oxygen therapy in breast cancer patients with late radiation toxicity (HONEY trial): a trial protocol using a trial within a cohort design

**DOI:** 10.1186/s13063-020-04869-z

**Published:** 2020-11-27

**Authors:** M. C. T. Batenburg, H. J. G. D. van den Bongard, C. E. Kleynen, W. Maarse, A. Witkamp, M. Ernst, A. Doeksen, T. van Dalen, M. Sier, E. J. P. Schoenmaeckers, I. O. Baas, H. M. Verkooijen

**Affiliations:** 1 Department of Radiation Oncology, University Medical Centre Utrecht, Heidelberglaan 100, 3584 CZ Utrecht, the Netherlands; 2 Department of Plastic, Reconstructive and Hand Surgery, University Medical Centre Utrecht, Utrecht, the Netherlands; 3 Department of Surgery, University Medical Centre Utrecht, Utrecht, the Netherlands; 4 Department of Surgery, Alexander Monro Ziekenhuis, Bilthoven, the Netherlands; 5 Department of Surgery, St. Antonius Ziekenhuis, Nieuwegein, the Netherlands; 6 Department of Surgery, Diakonessenhuis, Utrecht, the Netherlands; 7 Department of Surgery, Ziekenhuis Rivierenland, Tiel, the Netherlands; 8 Department of Surgery, Meander Medisch Centrum, Amersfoort, the Netherlands; 9 Department of Oncology, University Medical Centre Utrecht, Utrecht, the Netherlands; 10 Imaging Division, University Medical Centre Utrecht, Utrecht, the Netherlands

**Keywords:** Breast cancer, Radiotherapy, Hyperbaric oxygen therapy, Late toxicity, Trials within cohorts, Patient-reported outcomes

## Abstract

**Background:**

Breast cancer treatment with radiotherapy can induce late radiation toxicity, characterized by pain, fibrosis, edema, impaired arm mobility, and poor cosmetic outcome. Hyperbaric oxygen therapy (HBOT) has been proposed as treatment for late radiation toxicity; however, high-level evidence of effectiveness is lacking. As HBOT is standard treatment and reimbursed by insurers, performing classic randomized controlled trials is difficult. The “Hyperbaric OxygeN therapy on brEast cancer patients with late radiation toxicity” (HONEY) trial aims to evaluate the effectiveness of HBOT on late radiation toxicity in breast cancer patients using the trial within cohorts (TwiCs) design.

**Methods:**

The HONEY trial will be conducted within the Utrecht cohort for Multiple BREast cancer intervention studies and Long-term evaluation (UMBRELLA). Within UMBRELLA, breast cancer patients referred for radiotherapy to the University Medical Centre Utrecht are eligible for inclusion. Patients consent to collection of clinical data and patient-reported outcomes and provide broad consent for randomization into future intervention studies. Patients who meet the HONEY in- and exclusion criteria (participation ≥ 12 months in UMBRELLA, moderate/severe breast or chest wall pain, completed primary breast cancer treatment except hormonal treatment, no prior treatment with HBOT, no contraindications for HBOT, no clinical signs of metastatic or recurrent disease) will be randomized to HBOT or control group on a 2:1 ratio (*n* = 120). Patients in the control group will not be informed about participation in the trial. Patients in the intervention arm will undergo 30–40 HBOT treatment sessions in a high pressure chamber (2.4 atmospheres absolute) where they inhale 100% oxygen through a mask. Cohort outcome measures (i.e., physical outcomes, quality of life, fatigue, and cosmetic satisfaction) of the HBOT group will be compared to the control group at 3 months follow-up.

**Discussion:**

This pragmatic trial within the UMBELLA cohort was designed to evaluate the effectiveness of HBOT on late radiation toxicity in breast cancer patients using the TwiCs design. Use of the TwiCs design is expected to address issues encountered in classic randomized controlled trials, such as contamination (i.e., HBOT in the control group) and disappointment bias, and generate information about acceptability of HBOT.

**Trial registration:**

ClinicalTrials.gov. NCT04193722. Registered on 10 December 2019.

**Supplementary information:**

The online version contains supplementary material available at 10.1186/s13063-020-04869-z.

## Background

With increasing incidence and survival of breast cancer, and the therefore growing number of breast cancer survivors, long-term outcomes and side effects after breast cancer and breast cancer treatment have become increasingly important [1]. In most parts of the world, radiotherapy is part of the multimodality treatment of breast cancer in the majority of patients [1]. Radiotherapy reduces the risk of local recurrence and improves disease-free survival [2, 3]. However, it may also induce late radiation toxicity, including breast or chest wall pain, fibrosis, edema, impaired arm mobility, and decreased cosmetic outcome at least 12 months after radiation treatment.

One of the proposed treatment options for late radiation toxicity in breast cancer patients can be hyperbaric oxygen therapy (HBOT). HBOT induces neovascularization and stimulates collagen formation by fibroblasts [4]. Although HBOT is currently used in the treatment of late radiation toxicity in the breast and reimbursed by insurers, evidence of clinical effectiveness is limited [5, 6]. Also, HBOT has several side effects, such as (transient) myopia (12.8%), fatigue (14.0%), barotrauma (i.e., problems with clearing the ears due to the high pressure) (15.1%), or oxygen toxicity (0.003–1.7%) [5, 7, 8]. Oxygen toxicity is characterized by seizures, which will resolve after removal from the hyperbaric tank. Patients suffer from no additional consequences due to the oxygen toxicity and might even finish the other HBOT sessions [8]. Several small, non-randomized studies with limited follow-up have suggested a beneficial effect of HBOT in breast cancer patients, especially in terms of pain and arm mobility [5, 6].

Conducting randomized controlled trials (RCTs) with HBOT is challenging. First, patients with severe complaints may, when asked to participate in an RCT, refrain from participation because they do not want to be randomized to the control arm [9]. Also, participants might drop out after being randomized to the control arm, and obtain HBOT at their own initiative. An alternative trial design to overcome these issues is the trials within cohorts (TwiCs) design [10]. In TwiCs, the trial is nested in a prospective cohort study with regular outcome measurements. Eligible patients meeting the trial-specific inclusion criteria will be randomized to an intervention group or control group. Patients allocated to the intervention group will then be offered the intervention. The control group will not be informed about the trial. By using the cohort outcome measurements, outcomes in the intervention group are compared to outcomes in the control group.

In this study, we use the TwiCs design to investigate the effectiveness of hyperbaric oxygen therapy in comparison to usual care in breast cancer patients with late radiation toxicity.

## Methods

### Study design

This study will be performed within the UMBRELLA cohort [11]. In the prospective UMBRELLA cohort, all breast cancer patients referred for radiotherapy to the University Medical Centre Utrecht are eligible for inclusion. Currently, over 3300 patients are included and inclusion is ongoing. Upon inclusion, patients consent for (re)use of their clinical data and collection of patient-reported outcomes (PROMs) and they provide broad consent for randomization into future intervention studies [12].

**Fig. 1:**
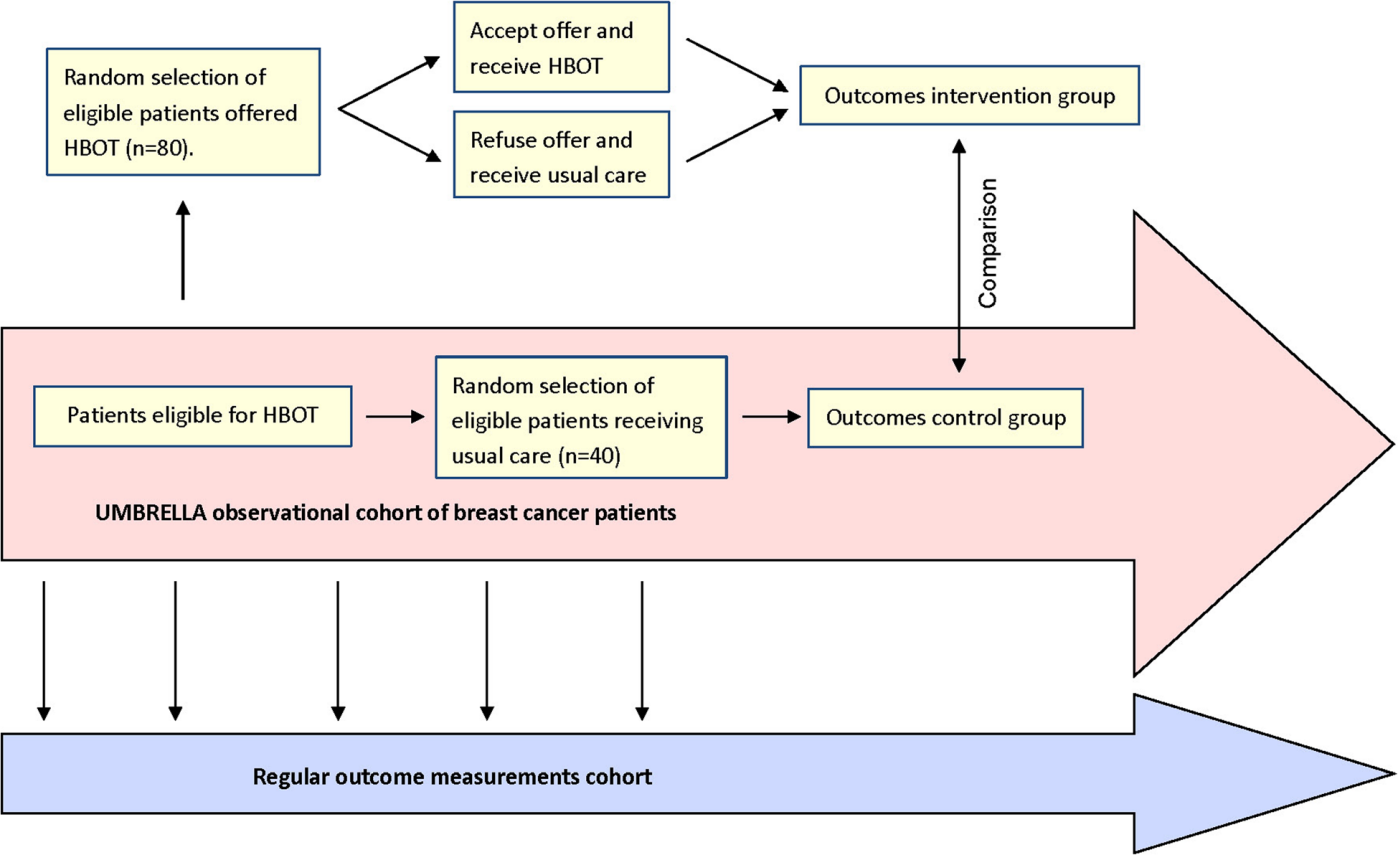
Design of the UMBRELLA HONEY trial; a trial within cohorts design (TwiCs design), figure adapted from Relton et al. [10]

The HONEY study follows the TwiCs design [10]. Within the UMBRELLA cohort, eligible patients (i.e., patients with late radiation toxicity), who consented for future randomization, will be identified as a sub-cohort for the HONEY trial. Patients from this sub-cohort will be randomized in a 2:1 ratio. Afterwards, patients allocated to the intervention arm will be offered HBOT, which they can accept or refuse (Fig. 1). Patients who refuse HBOT will receive usual care, but remain in the intervention arm. Patients who were allocated to the control arm will receive usual care and will not be informed about the trial. Their outcomes will be collected within the standard follow-up of the UMBRELLA cohort.

Patients with late radiation toxicity will be eligible for participation in the HONEY trial. In order to identify patients with late radiation toxicity, a self-developed late radiation toxicity questionnaire will be sent out to UMBRELLA participants who are > 12 months after the last radiotherapy fraction. The late radiation toxicity questionnaire consists of questions from different validated questionnaires. Breast and chest wall pain, social functioning, and other breast symptoms will be assessed with questions from the European Organisation for Research and Treatment of Cancer Quality of Life Questionnaire C30 (EORTC QLQ-C30), the breast-specific questionnaire EORTC QLQ-BR23, and Common Terminology Criteria for Adverse Events [13, 14]. In addition, specific questions were added by the researchers to assess possible late radiation toxicity in further detail and to evaluate eligibility criteria for the HONEY trial.

Eligibility criteria include self-reported breast pain or chest wall pain score in the late radiation toxicity questionnaire of 3 or 4 (on a scale of 1 to 4) and completed primary treatment for breast cancer (except endocrine treatment). Patients are ineligible when they were previously treated with HBOT, have contraindications for HBOT (e.g., (severe) COPD/asthma, pacemaker, morbid obesity, epilepsy in medical history, severe heart failure), and have current metastatic disease or recurrent breast cancer or when they are poor responders to UMBRELLA questionnaires (i.e., return of ≤ 2 questionnaires).

### Randomization and informed consent

In addition to the primary endpoints, other effects for the patients receiving hyperbaric oxygen therapy (i.e., tissue oxygenation, side effects of HBOT, physician-reported outcomes) will be important to evaluate. As a large effect was assumed, the sample size needed to demonstrate a significant effect was rather small. Therefore, in order to be able to answer secondary research questions, a 2:1 ratio for HBOT vs. control group randomization was applied to increase the size of the intervention arm.

A computer-generated randomization list with varying block sizes (*n* = 3–6) will be generated by an independent data manager prior to the first inclusion. Randomization will be stratified for time since inclusion in the UMBRELLA cohort (i.e., ≤ 2.5 years or > 2.5 years after start radiotherapy). The randomization list is linked to a specially designed inclusion database in Microsoft Access. The investigator has no access to the randomization list. After enrolment in the inclusion database, Microsoft Access will allocate patients to their respective treatment.

**Fig. 2:**
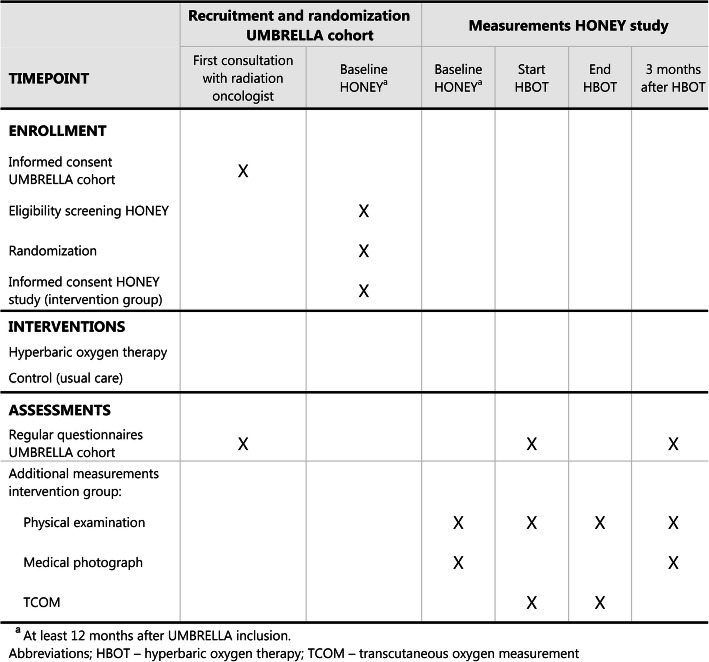
Schedule of enrollment, interventions, and assessments in the HONEY study

In accordance with the TwiCs design, patients randomized to the HBOT arm will be contacted by the investigator and offered to undergo HBO treatment. If they agree, they sign a second informed consent form in addition to the previously signed informed consent form of the cohort. Also, patients have the option to consent for the use of their clinical data for other studies on the same subject. In case patients drop out after providing informed consent, patients are asked for permission for the use of their clinical data in the trial. This trial does not involve collecting biological specimens for storage. The informed consent form is available from the corresponding author upon request. Patients who refuse the offer to undergo HBOT will receive treatment as usual, i.e., standard follow-up. Standard follow-up may entail physiotherapy, edema therapy, and/or analgesics, depending on the patient’s needs. Patients who are allocated to the control arm will not be informed about the HONEY trial, and undergo standard follow-up. For logistic reasons and planning of HBOT, patients will be recruited in batches (Fig. 2). After confirmation of diagnosis by a radiation oncologist, patients provide informed consent and will be referred for hyperbaric oxygen therapy.

### Hyperbaric oxygen therapy group

The combination of high pressure and 100% oxygen inhalation induces neovascularization and regeneration in the irradiated (hypoxic) tissue [4]. During HBOT, patients are seated in a hyperbaric chamber in which the pressure will be raised from 1.0 atmospheres absolute (ATA) to 2.4 ATA. Subsequently, 100% oxygen is given through a mask placed over nose and mouth for 20 min. One treatment session of HBOT is divided into 4 parts of maximum 20 min during which patients inhale 100% oxygen. In between these parts, there are small breaks without a mask, to decrease the risk on oxygen toxicity. After the oxygen sessions, the pressure will be decreased to 1.0 ATA.

To make sure patients are eligible for hyperbaric oxygen therapy, patients will be seen by a hyperbaric oxygen therapy physician. If patients are not “fit to dive” (e.g., in case of a respiratory tract infection) in between HBOT sessions or prior to HBOT, the HBO physician might decide to cancel a HBOT session to ensure patients’ safety. In case of missed HBO sessions, the HBO physician will decide whether effectivity of HBOT is endangered. Depending on judgment of the hyperbaric oxygen physician, the HBOT might be canceled or prolonged at the end.

HBOT consists of 30–40 hyperbaric oxygen sessions (i.e., one session of 2 h per day, 5 days/week). There are appointments with the HBO physician scheduled after 15 and after 30 HBO sessions, since the first effects of HBOT on late radiation toxicity will occur after 20–30 HBO sessions. Therefore, the patient and the HBO physician will decide together whether or not an additional 10 sessions will be valuable after 30 HBO sessions, depending on the effects achieved with HBOT so far. In between hyperbaric oxygen sessions, patients in the intervention group might still require edema therapy and physiotherapy or use analgesics (i.e., usual care). All concomitant care is permitted; the use of these treatments will be monitored.

### Control group

The patients randomized to the control group will not be notified about the UMBRELLA HONEY trial and will receive usual care. As usual care entails many different treatment options, including HBOT, patients in the control group will be monitored to evaluate the treatment they undergo for the late radiation toxicity.

### Primary and secondary endpoints

The primary endpoint of this study is the difference in proportion of patients with severe/moderate reported breast/chest wall pain between both groups at 3 months follow-up (Fig. 2). Upon inclusion, all patients will have moderate/severe pain, as this is an inclusion criterion. Self-reported pain is assessed through the late radiation toxicity questionnaire on a 4-point Likert scale (i.e., none/mild/moderate/severe). Self-reported pain is dichotomized into none/mild pain and moderate/severe pain.

Secondary endpoints include physical functioning, QoL, cosmetic outcome, physician-reported pain and radiation toxicity, tissue oxygenation, and side effects of HBOT. Physical functioning will be evaluated using the late radiation toxicity questionnaire, containing questionnaires on breast and arm edema, arm mobility, and breast fibrosis. QoL will be assessed by means of the EORTC QLQ-C30 and breast-specific questionnaire EORTC QLQ-BR23 [13]. In the UMBRELLA cohort, QoL is measured upon inclusion (before start radiotherapy), at 3 months, and every 6 months afterwards. Self-reported cosmetic outcome will be assessed using the BREAST-Q questionnaire [15, 16]. Depending on previous surgery, patients fill out a different module (mastectomy/breast conserving therapy/reconstruction) yearly within the UMBRELLA cohort. Side effects will be monitored using the MacFie classification [17].

### Additional measurements intervention group

Patients included in the intervention arm, who accepted to undergo HBOT, will visit the UMC Utrecht prior to the start of HBOT and 3 months after the last hyperbaric oxygen session (Fig. 2). The first visit is a combined visit to obtain informed consent, perform physical examination by a radiation oncologist to confirm diagnosis, and obtain a standardized digital photo for cosmetic outcome.

Physical examination includes breast and/or chest wall examination to assess the extent of baseline toxicity edema and fibrosis according to the Common Terminology Criteria for Adverse Events (CTCAE) version 4.03 [14]. The Patient and Observer Scar Assessment Scale (POSAS) will be used as a scar rating scale [18]. The extent of impaired arm mobility will also be assessed. Upon inclusion, auscultation of the heart and lungs and an ear exam will be performed to assess eligibility for HBOT.

Three months after the last hyperbaric oxygen session, patients will visit the UMC Utrecht again for physical examination and a medical photo.

#### Transcutaneous oxygen measurement

Shortly prior to the HBOT, transcutaneous oxygen measurement (TCOM) will be performed and repeated 3 months after the last HBOT session (Fig. 2). TCOM is a local and non-invasive measurement [19]. With 2–4 sensors on the skin, the diffused oxygen in the skin is measured. The temperature in the sensor is slightly increased during measurement, inducing vasodilatation. Oxygenation of tissue with late radiation toxicity will be compared before and after HBOT, and to the contralateral breast without late radiation toxicity.

#### Medical photograph

A medical photograph will be taken prior to the first HBOT session and 3 months after the last HBOT session. This digital photo will be judged by expert physicians (with different medical backgrounds) to assess cosmetic outcome. These physicians will be blinded for the moment the digital photo was taken (i.e., prior or after HBOT). In addition, the symmetry of the breast (in case of breast conserving surgery or breast reconstruction) will be assessed by the BCCT.core program [20].

### Data management and trial monitoring

Every 3 months, the trial proceeding is evaluated by the trial steering group. The trial steering group consists of the principal investigator, study coordinator, and supervising staff members of the UMC Utrecht. Daily coordination, recruitment of subjects, and inclusion of trial subjects are the responsibility of the study coordinator.

In addition, study progress and data management are evaluated by an independent trial monitor.

The trial monitor evaluates adherence of the data management plan, protocol adherence, and trial progress prior to the start of the trial, after inclusion of 5 patients, and yearly afterwards. At the end of the trial (i.e., after the last patient had the last visit to the UMC Utrecht), a closing visit will be scheduled. The data management plan encompasses detailed information on data collection and storage (Additional file 1). The independent trial monitor will report outcomes of the monitoring to the institutional review board. A Data Monitoring Committee was not considered as HBOT is a low-risk intervention. The trial sponsor played no part in the study design, writing of the report, or decision to submit the report for publication. Also, the trial sponsor will not play a part in the collection of data, study management, and data analysis.

Aggregated results of the trial will be reported to all UMBRELLA patients after analysis through the annual newsletter. No post-trial care is scheduled, as no long-term harm of HBOT is anticipated. During the trial, the physicians of trial patients will be informed about the participation. Trial results will be published after analysis. Any data required to support the trial protocol as well as trial data can be supplied by the corresponding author upon reasonable request.

### Sample size considerations

Since moderate/severe pain is an inclusion criteria, all patients will have moderate or severe pain upon inclusion. We assume that 3 months after the last hyperbaric oxygen session, the proportion of patients treated with HBOT suffering from moderate to severe pain will decrease to 30% [5]. Both control patients and patients who refuse HBOT will receive usual care. It is not expected that the offer of HBOT will influence the outcome at follow-up. Consequently, we assume that of the patients receiving usual care, at least 80% will be reporting moderate/severe pain at the same time point.

It is expected that 50% of the women in the HBOT arm, who will be offered HBOT, will accept and undergo the treatment. As such, in the intervention arm, the overall proportion of women reporting moderate to severe pain will be 55% (0.5 × 30% + 0.5 × 80%) and 80% in the control group. In line with the TwiCs design, the control patients are not informed about the HBO treatment. Consequently, there will be no refusal in the control arm.

The purpose of this study is to evaluate if HBOT is either similar or better than usual care. Therefore, a directional (i.e., one-sided) test will be used. To demonstrate a significant difference of 55% vs. 80% with a power of 80%, a one-sided alpha of 0.05, and an inclusion ratio of HBOT vs. control group of 2:1, we need 72 patients in the HBOT arm and 36 patients in the control group. However, drop-out might be expected. These are not patients who refuse participation, but drop-out for other reasons, such as patients who no longer wish to participate in the UMBRELLA cohort or patients who accept the offer of HBOT, but drop-out afterwards. In order to adjust for 10% drop-out, a total of 120 (80:40) patients will be enrolled in the UMBRELLA HONEY trial. Enrollment is expected to take 20 months.

### Data analysis

Outcomes of eligible patients who were randomly offered HBOT will be compared with eligible patients who were randomly selected from the control group. In case of non- or incomplete compliance with the intervention (i.e., patients not finishing all 30–40 HBO sessions), a worst-case analysis will be performed: dropped-out patients will be classified as non-responders. As part of the TwiCs design, non-compliance is only expected in the intervention group. In addition, patients in the control group may also undergo HBOT outside the trial setting. To account for the non-compliance in the intervention group and possible contamination in the control group, a Complier Average Causal Effect (CACE) analysis will be used in addition to the intention to treat analysis [21, 22]. In a CACE analysis, the group who accepted the HBOT will be compared to the control group who would have accepted the intervention if they had received the offer.

The primary outcome will be presented in absolute numbers and proportions. The primary outcome is defined as difference in proportion of patients with moderate/severe pain at 3 months follow-up per allocated treatment arm (i.e., intervention or control group). As pain is measured on a 4-point Likert scale, it will be dichotomized into no/mild pain and moderate/severe pain. Pain response is defined as decrease in pain from self-reported moderate/severe pain to no/mild pain. Differences in pain response will be compared by *χ*^2^ test. As secondary analysis, an unadjusted logistic regression analyses will be performed. In addition, as sensitivity analysis, the logistic regression analysis will be adjusted for age, time since radiotherapy, radiotherapy dose, and smoking. There will potentially be missing data. Assuming that missing values are missing at random, multiple imputation by chained equations for the primary analysis will be used to replace missing values, using 20 imputed datasets [23–26]. In addition, complete case analysis will be performed as sensitivity analysis. Toxicity will be presented as the overall incidence of grade 2–4 toxicity. QoL outcomes will be evaluated at 3 time points: baseline in the UMBRELLA cohort, prior to HBOT, and at follow-up. To account for the intra-subject correlation over time, a mixed model for repeated measurements will be used. In the model, a random intercept and random linear time effect and an autoregressive covariance structure of the first order (AR1) (assuming that the correlation systematically decreases with increasing distance between time points) will be included [27]. Also, fixed effects for treatment arm and an interaction between time and treatment arm will be included, as well as characteristics with imbalances as previously described. Differences with a *P* value < 0.05 will be considered statistically significant.

Given the relatively small sample size of the study, we will not be performing an interim analysis, as it is very unlikely that we will see a highly significant effect of HBOT justifying early stopping of the trial. Also, there is ample clinical evidence that HBOT is safe and associated with a very small risk of mild side effects. Therefore, no side effects are expected that might lead to early termination of the study. Consequently, no interim analysis was planned for this study.

### Ethical approval

Ethical approval was obtained for both the UMBRELLA study (including the TwiCs infrastructure) and the HONEY trial (protocol version 3, d.d. 23 July 2019) from the institutional review board of the UMC Utrecht. The UMBRELLA study was published under NCT02839863 [11] and the HONEY study under NCT04193722 on ClinicalTrials.gov.

## Discussion

The HONEY study aims to assess the effectiveness of hyperbaric oxygen therapy on late radiation toxicity in breast cancer patients. HBOT is by some considered as a potentially curative treatment for late radiation toxicity in breast cancer patients. In a study by Teguh et al., the effects of 40 sessions with HBOT of 57 patients with late radiation toxicity were assessed. Pain score was assessed by means of the NRS score [5]. An improvement of ≥ 1 point after immediately after treatment was seen in 81% of the patients. Also, a significant improvement of self-reported arm mobility, swelling of the breast and arm, skin problems, oversensitivity of the breast, and pain in arm or shoulders immediately post-HBOT was observed (assessed by EORTC BR23). However, limitations of this study are the absence of a control group and the small sample size.

In a prospective study, Carl et al. compared 32 breast cancer patients treated with HBOT with 12 patients who refused to undergo HBOT. The median number of HBOT sessions was 25 and ranged from 7 to 60 sessions, since treatment was stopped when 3 consecutive sessions did not result in improvement. Late radiation toxicity was assessed by means of the LENT-SOMA score, a score conducted through physical examination [28]. In this small, non-randomized study, a significant reduction in pain, edema, and erythema of HBOT patients in comparison to non-treated patients was seen. In conclusion, evidence is limited and a randomized trial is needed.

Currently, HBOT is reimbursed by insurers for symptoms of late toxicity, complicating evaluating its efficacy in a classic RCT. In a classic RCT comparing usual care to HBOT, patients allocated to the control arm may be disappointed and report worse outcomes, leading to disappointment bias. Also, patients might drop out after being randomized to the control group and undergo HBOT at their own initiative. A classic randomized controlled trial by Teguh et al. randomized 19 patients with oropharyngeal and nasopharyngeal cancer for HBOT or control group (usual care) immediately after radiotherapy [9]. Prior to HBOT, self-reported complaints, such as dry mouth, were significantly higher for control patients than HBOT patients, despite randomization. Also, the study was stopped prematurely due to slow accrual, leading to only 19 patients eligible for analysis.

An alternative is the sham-controlled trial, in which patients in the control group undergo 40 sham sessions in a hyperbaric oxygen chamber with only slightly elevated air pressure and inhale normal air instead of 100% oxygen. From an ethical perspective, it may be undesirable to expose patients to a high burden, i.e., 40 2-h sessions, unnecessary. Previously in a trial by Clarke et al., 150 patients with radiation proctitis were randomized to HBOT or sham-controlled group [29]. To overcome the ethical issue of the burden for the control group, patients were crossed-over after 40 sham sessions. Consequently, it is impossible to obtain long-term follow-up results for the control patients with this design.

The TwiCs design aims to overcome problems, such as disappointment bias, slow accrual, and dropout in the control group, since patients in the control group are unaware of being a control. Upon inclusion in the UMBRELLA cohort, patients consent to future randomization, and after the trial, the entire cohort will be informed about the results obtained in the HONEY trial. Also, since the HONEY trial participants are also UMBRELLA participants, follow-up can continue for years after completion of HBOT.

A limitation of the TwiCs design is the dependency of data collected within the cohort. In order to assure that control patients remain unaware of their participation in the trial, it is for example not possible to perform additional (invasive) physical measurements on these patients. Also, eligibility for the trial is assessed by means of a self-reported questionnaire on late radiation toxicity and not physical examination, in contrast to current practice. However, literature suggests that patient-reported late radiation toxicity does not underestimate late side effects reported by physicians [30].

In summary, the HONEY trial is a pragmatic trial in accordance with the TwiCs design. The HONEY trial aims to evaluate the efficacy of HBOT in breast cancer patients with late radiation toxicity.

## Trial status

Ethical approval was obtained in August 2019 (protocol version 3; 23 July 2019). Recruitment started in November 2019 and is still ongoing. Recruitment is expected to be completed in September 2023.

## Supplementary Information

**Additional file 1.** Data management plan.

## Data Availability

Not applicable, since recruitment is ongoing and no data were yet obtained.
