# Supplementary material for: Assessing the effect of hyperbaric oxygen therapy in breast cancer patients with late radiation toxicity (HONEY trial): a trial protocol using a trial within a cohort design
Source: Trials. 2020 Nov 27;21:980. doi: 10.1186/s13063-020-04869-z (PMC7694912; doi:10.1186/s13063-020-04869-z)
Supplement: Supplementary file 1 — Additional file 1. Data management plan. [file 13063_2020_4869_MOESM1_ESM.pdf]

DATA MANAGEMENT PLAN

(basic questions)

GENERAL INFORMATION

Study title:	The effect of Hyperbaric OxygeN therapy on breast cancer patients with late radiation toxicity
Acronym:	UMBRELLA HONEY trial
Kind of study:	☒ WMO research<br>☐ Non-WMO – prospective data collection<br>☐ Non-WMO – (re-)use of existing healthcare/research data<br>☐ Biobank
Date:	6-3-2019
Principal investigator:	Prof. H.M. Verkooijen, MD, PhD
Other investigator(s):<br>(enter your name here if you are not the principal investigator)	M.C.T. Batenburg, MD, PhD student<br>H.J.G.D. van den Bongard, MD, PhD, radiation oncologist<br>A. Witkamp, MD, PhD, surgical oncologist<br>I.O. Baas, MD, PhD, oncologist<br>W. Maarse, MD, PhD, plastic surgeon
Department:	Radiotherapy
Sponsor:<br>(Dutch: Verrichter)	University Medical Center Utrecht
Name of datamanager / KC:	Saskia van Amelsvoort

1. What data will be collected?

- Medical and socio-demographical information, such as age, gender, primary tumor, breast cancer treatment (part of UMBRELLA cohort)
- Quality of life, fatigue, cosmetic outcome, late radiation toxicity complaints will be collected for both the HBOT group and the control group. Data about late radiation toxicity and cosmetic outcome will be collected both patient reported and physician reported by means of questionnaires and physical examination. Physician reported outcomes will only be collected in the HBOT group.
- Skin oxygenation (in the HBOT group)
- Side-effects of HBOT (in the HBOT group)
- Patient reported experience with HBOT (in HBOT group)

2. How will data be collected or created?

Medical history and collected data in the context of the UMBRELLA cohort study will be collected and stored as described in the UMBRELLA protocol.

Additional data about late radiation toxicity will be collected through a questionnaire. An additional physical examination will be performed twice to assess late radiation toxicity (according to CTCAE criteria) and cosmetic outcome (according to POSAS score). A transcutaneous oxygen measurement will be performed to asses skin oxygenation. Cosmetic outcome will also be evaluated through a medical photograph.

3. Do you need informed consent or research subject/patients according to laws and regulations? If yes, how is this or will this be arranged?

Yes, informed consent is needed.

Patients will be randomly selected based on the late radiation toxicity questionnaire. Potential eligible patients will be determined by the investigator checking the inclusion criteria. Patients randomly selected to be offered HBOT will receive an invitation letter by mail or on paper depending on a patients preference in the UMBRELLA cohort. The invitation letter includes the informed consent brochure and an informed consent form. All patients will be contacted by phone to gauge interest for the offered HBOT and, if interested, to explain further details. For eligible patients who are interested, an appointment will be made for and informed consent visit. During this visit informed consent will be signed.

4. How and where will data be stored and backed up during research? And for how long?

Collected data in de UMBRELLA HONEY trial will be stored coded in a special designed UMBRELLA HONEY Castor database. The database will be hosted on a secure multiprocessor server with the infrastructure, configuration and licences that are consistent with current norms and laws to ensure safe and secure data storage and processing. Hardcopy questionnaires will be physically stored in a secure archive of the UMC Utrecht.

Data collected as part of standard HBOT and transcutaneous oxygen measurements will be collected and stored in the same Castor database. All data acquired in the study will be stored for a maximum of 15 years after the end of the UMBRELLA HONEY study. After this period, all acquired data will be destroyed.

5. How will you manage access and security?

The UMBRELLA study USR number will be used. A separate identification log only containing USR numbers with corresponding patient identifiers (name, date of birth, sex and date of inclusion), is stored on a different secured server to prevent the possibility to directly match study data with patient identifiers.

The participant's name and personal data will remain confidential and will not be published in any way. However, the sponsor's monitor or representative and regulatory representatives (FDA and/or European Communities EU Notified Body Representatives), auditors and inspectors may have access to medical files in order to verify authenticity of data collected.
